# Diabetic fetopathy associated with bilateral adrenal hyperplasia and ambiguous genitalia: a case report

**DOI:** 10.1186/1752-1947-2-251

**Published:** 2008-07-25

**Authors:** Patou Tantbirojn, Mana Taweevisit, Suchila Sritippayawan, Boonchai Uerpairojkit

**Affiliations:** 1 Department of Obstetrics and Gynecology Faculty of Medicine, Chulalongkorn University, Rama IV road, Bangkok 10330, Thailand; 2 Department of Pathology, Faculty of Medicine, Chulalongkorn University, Rama IV road, Bangkok 10330, Thailand

## Abstract

**Introduction:**

Many fetal malformations can occur because of maternal diabetes. However, ambiguous genital organs have never been reported as an associated finding in the literature. This is the first report of associated ambiguous genital organ and bilateral adrenal hyperplasia in a case of diabetic fetopathy.

**Case presentation:**

A 19-year-old Thai primigravida with familial history of diabetes mellitus (DM) was diagnosed as having gestational DM type 2, based on 100 g oral glucose tolerance test, and was poorly controlled with insulin injections. Delayed targeted ultrasonography at 28 weeks gestation revealed multiple fetal anomalies. The woman underwent low transverse cesarean section at 30 weeks gestation due to preterm labor and transverse lie. The newborn with ambiguous genitalia was delivered but expired after birth. Autopsy findings revealed alobar holoprosencephaly, a prominent forehead, hypotelorism, an absent nose, absent bilateral ears, median cleft lip and palate, preaxial polydactyly of the right hand, accessory spleens, single umbilical artery, markedly enlarged adrenal glands and ambiguous external genitalia The subsequent fetal chromosomal study revealed 46,XX.

**Conclusion:**

We describe a case of diabetic fetopathy with classic facial malformation and preaxial hallucal polydactyly which has been proposed as a marker of diabetic embryopathy. Bilateral adrenal hyperplasia with ambiguous genitalia, an uncommon associated anomaly, was also identified. It is controversial whether adrenal hyperplasia can be a novel feature of diabetic fetopathy or just a coincidental finding. Further observation and adequate investigation are needed in such cases.

## Introduction

It is well known that maternal diabetes, type 1 or type 2 including gestational diabetes, increase the risk of congenital malformations [1]. The most frequent types of malformations involve the central nervous, cardiovascular, gastrointestinal, genitourinary, and skeletal systems [2]. Although in the present century, there have been significant improvements in the management and outcome of diabetic women and infants and most neonatal problems have declined, nevertheless diabetes-associated anomalies still remain a major health problem in Thailand [3]. While many fetal malformations occur as a result of maternal diabetes, ambiguous genital organs are not included. Overall, congenital adrenal hyperplasia is the most frequent cause of ambiguous genitalia in newborns and is the most common cause of virilization of female external genitalia [4]. The association of diabetic embryopathy with bilateral adrenal hyperplasia has never been reported in the English medical literature, based on online medical searching. We describe a case of maternal diabetes with multiple fetal anomalies and associated bilateral fetal adrenal hyperplasia.

## Case presentation

A 19-year-old Thai primipara presented at our outpatient department for routine antenatal care at 18 weeks gestation. She had no history of any underlying disease and denied family history of genetic abnormality or congenital adrenal hyperplasia. Her mother had a history of diabetes mellitus (DM), unknown type. Her 14-year-old brother also had a history of DM type 1 and was treated with insulin injections. However, the woman had never been tested for diabetes. 50 g glucose challenge test was performed at the first antenatal visit due to her family history of diabetes, showing 234 mg/dl, thus a 100 g oral glucose tolerance test was done. The result were 118, 227, 234 and 173 mg/dl at fasting, 1 hour, 2 hours and 3 hours post 100 g glucose ingestion, respectively. Gestational DM type 2 was diagnosed and the woman was treated with insulin injections. She had poor compliance with the treatment and had rather poor diabetic control. HbA1C level was high and the patient refused to be admitted as an inpatient to control her diabetes. Targeted ultrasonographic examination was postponed several times due to poor compliance and was finally carried out at 28 weeks gestation, revealing a single viable fetus in unstable lie with polyhydramnios and multiple fetal anomalies, consisting of holoprosencephaly, hypotelorism, median cleft lip and polydactyly of the right hand. Prenatal chromosomal testing was offered, but the woman refused and counseling of the fetal prognosis was subsequently provided to the couple. Low transverse cesarean section was performed at 30 weeks of gestation due to fetal transverse lie when she had preterm labor. The newborn with ambiguous genitalia was delivered with Apgar scores at 1 and 5 minutes of 4 and 3 respectively and was handed to the waiting neonatologist in the operative room. The newborn finally expired due to respiratory failure eighteen minutes after birth. The subsequent fetal chromosomal study revealed 46,XX.

**Figure 1:**
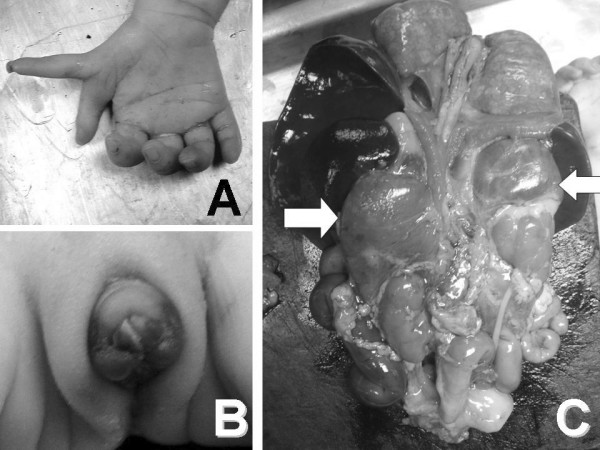
**A) Preaxial polydactyly of the right hand.** B) Ambiguous external genitalia. C) Enlarged adrenal glands.

**Figure 2:**
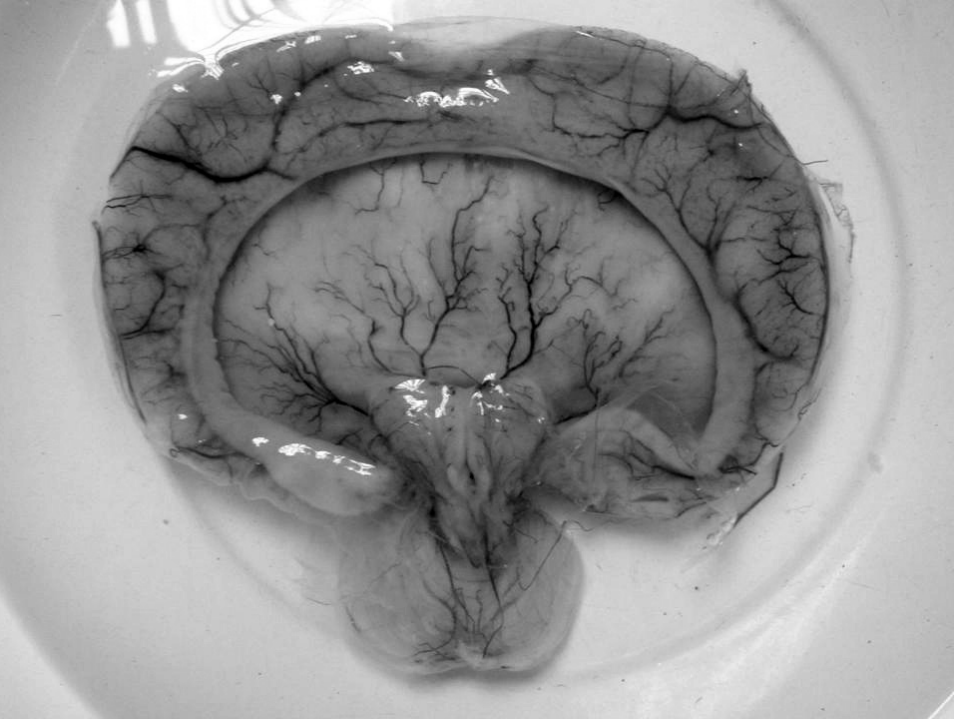
Alobar holoprosencephaly.

An autopsy was performed four hours after death, showing an ambiguous sex infant weighing 1,720 grams and measuring 38 cm in length. The head, chest and abdominal circumference were 40, 20 and 20 cm respectively. Multiple facial anomalies were seen, consisting of a prominent forehead, hypotelorism, an absent nose, absent bilateral ears, and a median cleft lip and palate. Preaxial polydactyly of the right hand (figure 1A) and ambiguous external genitalia, with clitoral hypertrophy and hyperpigmentation (figure 1B), were observed. On examination of the internal organs, the uterus and both ovaries were in the normal anatomical position. There were two small accessory spleens identified at the splenic hilum. Markedly enlarged adrenal glands were seen (figure 1C) and serial sectioning revealed a homogenous tan-brown cut surface. The other internal organs were unremarkable. Examination of the brain revealed a single dilated ventricular cavity with no falx, corpus callosum or interhemispheric fissure (figure 2) and hydrocephalus with cerebrospinal fluid of 200 ml. Examination of the placenta and umbilical cord revealed only a single umbilical artery with no other significant abnormality.

## Discussion

Malformations in infants of diabetic mothers are well known. Infants born to insulin-dependent diabetic mothers have a 2- to 3-fold increased risk of congenital malformations and spontaneous abortion. The prevalence of major congenital malformations in the offspring of affected women is approximately 4–8% [2]. The exact teratogenic mechanism of this malformation is unknown. On the basis of a recent animal study by Loeken [5], increased glucose delivery to embryos results in activation of pathways that are stimulated by high glucose, such as the hexosamine biosynthetic pathway, or hypoxia; therefore, oxidative stress in embryos is increased. Because oxidative stress may impair embryo gene expression and consequent apoptosis, or disturb organogenesis, fetal malformation eventually occurs.

The most frequently noted malformations are anomalies of the cardiovascular, genitourinary and central nervous system. In addition, infants with caudal dysplasia, femoral hypoplasia and unusual facial features are born more frequently to diabetic mothers [6].

Multiple anomalies were identified in this case. Alobar holoprosencephaly with midline facial defects (hypotelorism, arhinia, median cleft lip and palate) and bilateral auricular atresia are compatible with diabetic fetopathy. Interestingly, preaxial polydactyly or hallucal polydactyly is also observed in this case. This condition was reported by some authors as a distinctive anomaly in diabetic embryopathy and might be a useful clinical marker for the diagnosis of diabetic embryopathy [7,8]. We also identified other unusual associated anomalies in this case, such as accessory spleens and a single umbilical artery. However, the most striking associated anomaly was bilateral adrenal hyperplasia with virilization of female external genitalia.

Congenital adrenal hyperplasia is the most common genetic cause of ambiguous genitalia in female newborns. Other causes of fetal female virilization during pregnancy include maternal progesterone, androgen or androgenic drug administration, and androgen production by maternal adrenal or ovarian tumors. As these factors were not identified in this case, and given the additional finding of markedly enlarged adrenal glands, adrenal hyperplasia was most likely to be the cause of the ambiguous genitalia.

In general, congenital adrenal hyperplasia is an autosomal recessive disease, caused by one of five different enzymatic defects in cortisol biosynthesis from cholesterol. Clinical manifestations are related to the degree of cortisol deficiency or aldosterone deficiency or a deficiency of both. Deficiency of 21-hydroxylase enzyme accounts for more than 90% of cases [9]. Androgen levels are increased and manifested in the female as clitoral hypertrophy, labial fusion, and sometimes scrotalization of the labia majora. Unfortunately, the investigation of specific precursor metabolites was not performed in this case, so the definite type of adrenal hyperplasia was unknown. Further genetic studies to identify specific gene mutations such as CYP21 (6q21.3) or CYP11B1 (8q24) loci may have a role, but it is not generally available. It is well documented that congenital hyperplasia may be the cause of hyperglycemia or diabetes [10], but hyperglycemia cannot explain the pathogenesis of adrenal hyperplasia in this case. It is controversial whether adrenal hyperplasia can be a novel feature of diabetic fetopathy or just a coincidental finding. Further observation and investigation are needed in such cases.

## Conclusion

We describe a case of diabetic fetopathy with classic facial malformation and preaxial hallucal polydactyly which has been proposed as a marker of diabetic embryopathy. Adrenal hyperplasia with ambiguous genitalia was also identified as an uncommon associated anomaly at autopsy.

## Competing interests

The authors declare that they have no competing interests.

## Authors' contributions

PT was involved in conception of the case report, autopsy, data collection, review of literature and writing the manuscript. MT participated in autopsy and data collection. SS and BU participated in prenatal ultrasonographic diagnosis and data collection. BU also helped to draft the manuscript. All authors read and approved the final manuscript.

## Consent

Written informed consent was obtained from the parent for the publication of this case report and any accompanying images. A copy of the written consent is available for review by the Editor-in-Chief of this journal.
